# Data Analytics for Predicting COVID-19 Cases in Top Affected Countries: Observations and Recommendations

**DOI:** 10.3390/ijerph17197080

**Published:** 2020-09-27

**Authors:** Abdelrahman E. E. Eltoukhy, Ibrahim Abdelfadeel Shaban, Felix T. S. Chan, Mohammad A. M. Abdel-Aal

**Affiliations:** 1 Systems Engineering Department, King Fahd University of Petroleum and Minerals, Dhahran 31261, Saudi Arabia; Abdelrahman.eltoukhy@kfupm.edu.sa (A.E.E.E.); mabdelaal@kfupm.edu.sa (M.A.M.A.-A.); 2 Faculty of Engineering, Helwan University, Helwan 11795, Egypt; 3 Department of Industrial and Systems Engineering, The Hong Kong Polytechnic University, Hong Kong, China; f.chan@polyu.edu.hk

**Keywords:** COVID-19, pandemic, data analytics, neural network

## Abstract

The outbreak of the 2019 novel coronavirus disease (COVID-19) has adversely affected many countries in the world. The unexpected large number of COVID-19 cases has disrupted the healthcare system in many countries and resulted in a shortage of bed spaces in the hospitals. Consequently, predicting the number of COVID-19 cases is imperative for governments to take appropriate actions. The number of COVID-19 cases can be accurately predicted by considering historical data of reported cases alongside some external factors that affect the spread of the virus. In the literature, most of the existing prediction methods focus only on the historical data and overlook most of the external factors. Hence, the number of COVID-19 cases is inaccurately predicted. Therefore, the main objective of this study is to simultaneously consider historical data and the external factors. This can be accomplished by adopting data analytics, which include developing a nonlinear autoregressive exogenous input (NARX) neural network-based algorithm. The viability and superiority of the developed algorithm are demonstrated by conducting experiments using data collected for top five affected countries in each continent. The results show an improved accuracy when compared with existing methods. Moreover, the experiments are extended to make future prediction for the number of patients afflicted with COVID-19 during the period from August 2020 until September 2020. By using such predictions, both the government and people in the affected countries can take appropriate measures to resume pre-epidemic activities.

## 1. Introduction

By January 2020, the COVID-19 outbreak that originated in China has spread globally, with the number of infected persons rising to 6,479,495 and a fatality of about 383,015 persons (https://www.worldometers.info/coronavirus. Last accessed 3 June 2020). With the global spread of this infectious disease, the World Health Organization (WHO) designated it as a pandemic. Besides the personal tragedies and casualties brought by this pandemic, the economic implications of this pandemic are significant. Most of the affected countries locked their borders and ordered closure of factories, restaurants, big malls, and clubs. Consequently, the world is suffering from an economic recession as the global economic losses are estimated to approach USD 23 trillion (the Economist, ‘‘Covid carnage,” 21 March 2020). This dire situation motivates researchers to conduct research on COVID-19 focusing on two main areas: medicine and engineering.

In the area of medicine, most of research has focused on understanding the symptoms of COVID-19 [1], characterizing it [2], and finally estimating its incubation periods [3]. Literature survey shows that the incubation period of the viral infection ranges from 4 to 14 days and many patients are asymptomatic [4]. This disease has a high infection rate; thus, it is very important to accurately predict and estimate the number of people affected by COVID-19. This motivates researchers to focus on another research area, the engineering aspect. This area is concerned with the prediction of COVID-19 cases. In the literature, there are two main methods used for prediction. The first method is the statistical and mathematical modeling, whereas second method is data analytics.

As for statistical methods, they have been applied to predict the COVID-19 cases in different countries like Italy [5,6], Iran [7,8], Spain and France [9]. Beside the statistical methods, the mathematical modeling and simulation have been utilized to predict the new COVID-19 cases in China [10] and Saudi Arabia [11]. Moreover, Papastefanopoulos et al. [12] have compared the accuracy of six time-series forecasting approaches, namely, ARIMA, Holt–Winters additive model (HWAAS), TBAT, Facebook’s Prophet, DeepAR, and N-Beats, in predicting the progression of COVID-19. Similarly, Hernandez-Matamoros et al. [13] have developed an ARIMA model to predict the spread of the virus, while considering some factors like the population and the number of infected cases. There are other studies that focus on collecting and analyzing posts related to COVID-19 from social media sites [14,15,16]. This is because the keyword search trends related to COVID-19 on search engines proved to be tremendously helpful in predicting and monitoring the spread of the virus outbreak. Although statistical and mathematical modeling and simulation have been widely adopted, they cannot consider a massive amount of data. Hence, the number of COVID-19 cases is poorly predicted. This drawback can be avoided by using the data analytics method.

Indeed, data analytics has infrequently been applied to the study of COVID-19, as few studies have been reported in the literature. For example, Chen et al. [17] have utilized data analytics to predict the number of COVID-19 cases to avoid overwhelming hospital capacity in Taiwan. Zhou et al. [18] have coupled Geographic information system (GIS) and data analytics together to identify the infection network of COVID-19. Additionally, machine learning and artificial intelligence tools have been utilized to develop COVID-19 prediction approaches. Wieczorek et al. [19] have developed a forecasting model for COVID-19 new cases based on the deep architecture of Neural Network using NAdam training model. Pinter et al. [20] have developed a hybrid machine learning approach to forecast COVID-19 cases in Hungary. For extensive study and more details about the forecasting approaches for COVID-19, the interested readers are referred to the work by Bragazzi et al. [21] who review the potentials of applying artificial intelligent and big data based approaches in predicting and managing the COVID-19 Pandemic outbreak. The pitfall of the previous studies is that they have focused on historical data of confirmed COVID-19 cases, while some studies have considered some factors like temperature and patient sex. Indeed, many other important external factors that affect the spread of the disease have been completely ignored. These important factors include population, median age index, public and private healthcare expenditure, air quality as a CO_2_ trend, seasonality as month of data collection, number of arrivals in the country/territory, and education index. This results in an inaccurate prediction of the number of COVID-19 cases.

The drawback in the previous studies has motivated us to conduct this study with the objective of predicting the number of COVID-19 cases while simultaneously considering the historical data of patients with COVID-19, and most of the external factors that affect the spread of the virus. To consider these massive data, data analytics has been utilized to develop a nonlinear autoregressive exogenous input (NARX) neural network-based algorithm. To demonstrate the efficiency of the developed algorithm, experiments have been conducted to predict the number of COVID-19 cases in top five affected countries in each continent.

The remainder of the study is organized as follows. In Section 2, we briefly present the literature review, whereas the research gap and contribution are presented in Section 3. The main procedure of the NARX neural network-based algorithm is described in Section 4. Section 5 and Section 6 present the results of the experiments and conclusions of the study, respectively.

## 2. Literature Review

### 2.1. Bibliographic Summary for COVID-19

**Figure 1:**
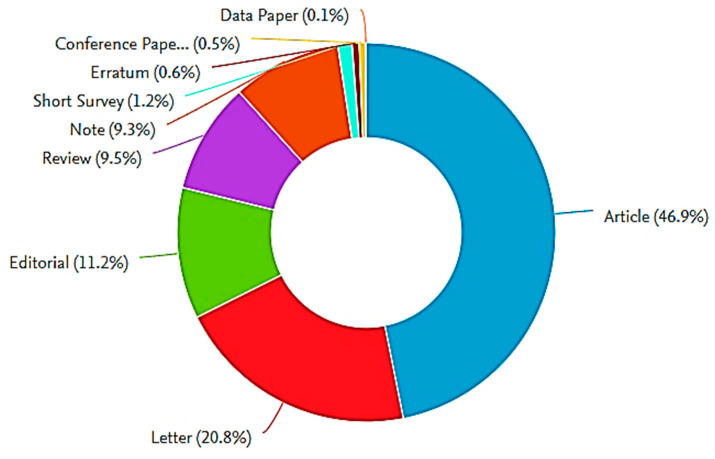
Types of documents published on COVID-19 disease (from Scopus Database).

Before investigating the literature on COVID-19, we conducted a brief bibliographic search about COVID-19 for two purposes. Firstly, to find out the number of research works published on COVID-19 and secondly, to identify the different research areas focusing on COVID-19. For those purposes, we used some keywords like COVID-19, novel coronavirus, and Hubei pneumonia. It was found that more than 8000 research documents have been published on this topic. Figure 1 shows the different types of research documents published on COVID-19. By looking at Figure 1, it can be observed that the vast majority of published works are in the form of journal articles, whereas a small number of research works have appeared as conference papers. This is because most conferences have been canceled due to the outbreak of the COVID-19 pandemic [22].

**Figure 2:**
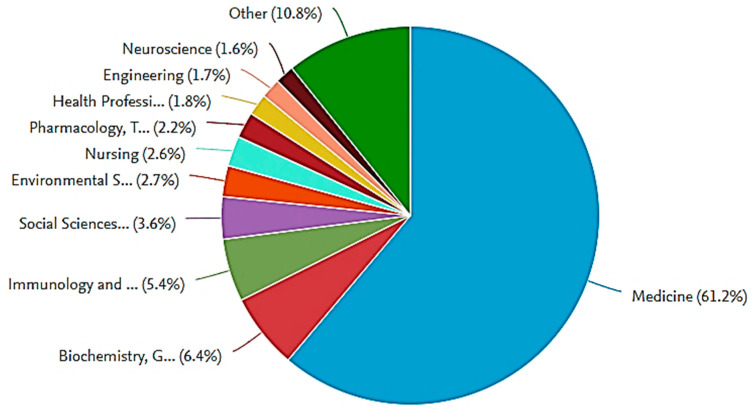
Classifications of COVID-19 publications based on the subject area (Scopus database).

Bibliographic search is then continued to identify the different research areas focusing on COVID-19. The findings are presented as a pie chart in Figure 2. As COVID-19 is a novel disease, it is noticed that the majority of these research works (around 61%) has focused on medicine, whereas the rest are distributed in different areas, like biochemistry, social sciences, and engineering. These research areas are discussed in the next section.

### 2.2. COVID-19 Related Works

In this section, we discuss the different research areas that have considered COVID-19, including medicine and engineering.

In the field of medicine, most of the early research on COVID-19 is focused on understanding the symptoms of the disease [1], characterizing it [2], and finally estimating its incubation periods [3]. In addition, Wang et al. [23] have reported that the elderly are more likely to die from COVID-19, because of their underlying comorbidities [24]. However, the virus attacks not only the elder people but also children [25]. This means that everybody can be infected by COVID-19. Moreover, Zhuang et al. [8] have showed that people infected with COVID-19 are asymptomatic in many cases.

COVID-19 has a long incubation period of 4 to 14 days, and in many cases, patients are asymptomatic [4]; thus, it has a high infection rate. Therefore, it is of great importance to predict and estimate the number of people affected by COVID-19. This motivates researchers to focus on the engineering aspect of this disease, that is, the prediction of COVID-19 cases. Usually, prediction can be conducted using traditional statistical methods. For example, Remuzzi and Remuzzi [5] and Tuite et al. [6] have utilized the statistical methods to predict the number of COVID-19 cases in Italy. Similarly, the number of COVID-19 cases has been predicted in different countries/territories such as, Iran [7,8], Spain, and France [9].

Beside the statistical methods as mentioned above, the mathematical modeling and simulation including Logistic Growth and Susceptible-Infected-Recovered (SIR-model) have been utilized to predict the new COVID-19 cases in China [10] and Saudi Arabia [11]. Moreover, Papastefanopoulos et al. [12] have investigated and compared the accuracy of six time-series forecasting approaches, namely, ARIMA, Holt–Winters additive model (HWAAS), TBAT, Facebook’s Prophet, DeepAR, and N-Beats, in predicting the progression of COVID-19. In a similar work, Hernandez-Matamoros et al. [13] have developed an ARIMA model to predict the spread of the virus. The developed model consists of ARIMA parameters, including the population of the country, the number of infected cases, and polynomial functions. Ivorra et al. [26] have proposed a new mathematical model for predicting the spread COVID-19 outbreak in China. The proposed model, θ-SEIHRD model, considers a fraction θ of detected cases over the realized total infected cases.

There are other studies that focus on collecting and analyzing posts related to COVID-19 from social media sites. This is because keyword search trends related to COVID-19 on search engines proved tremendously helpful in predicting and monitoring the spread of the virus outbreak. Qin et al. [14] developed a prediction technique based on the lagged series of social media search indexes to forecast the number of new suspected COVID-19 cases. The considered social media search indexes include common COVID-19 symptoms such as dry cough, fever, pneumonia, etc. In another study by Li et al. [15], the daily trend data related to specific keyword search such as “coronavirus” and “pneumonia”, has been acquired from Google Trends, Baidu Index, and Sina Weibo Index search engines to investigate and monitor new COVID-19 cases. Li et al. [16] have collected data on the posts related to COVID-19 that are posted by Chinese users on Weibo using an automated Python programming script. The collected data have been analyzed quantitatively and qualitatively in order to recognize trends and characterize key themes. Other applications using social media to predict COVID-19 cases have been reported by Shen et al. [27] and Ayyoubzadeh et al. [28]

The major drawback of statistical methods and mathematical modeling is their inability to consider massive amounts of data. This leads to poor prediction of the number of COVID-19 cases. This drawback can be avoided by using data analytics, which is explained in the next section.

### 2.3. Data Analytics

Data analytics is one of the efficient tools in discovering the relationships, trends, and other useful information existing in a body of data. The number of data analytics tools is large. Among these tools, the neural network is one of the most efficient tools in uncovering the relationship between an output (i.e., response) and multiple inputs (i.e., indicators) [29,30]. This efficiency has been applied in handling different applications, including stock price forecasting in the financial industry [31], flight delay prediction in aviation industry [32,33,34], organ prediction in healthcare sector [35], and demand forecasting in the railway industry [36].

These previous studies reveal the importance of data analytics for prediction purposes. This motivates researchers to adopt data analytics in the domain of COVID-19. For example, Chen et al. [17] utilized data analytics to predict the number of COVID-19 cases to avoid overwhelming hospital capacity in Taiwan. The pitfall of this research work is that it has only focused on historical data of the number of COVID-19 cases while considering a limited number of factors, like travel and occupation. Another research work by Zhou et al. [18] coupled Geographic information system (GIS) and data analytics together to identify the infection network of COVID-19. Additionally, machine learning and artificial intelligence tools have been utilized by many studies to develop COVID-19 prediction approaches. Wieczorek et al. [19] have developed a forecasting model for COVID-19 new cases based on the deep architecture of Neural Network using NAdam training model. However, the pitfall of this study is the focus on one dataset, called total number of confirmed COVID-19 cases, while overlooking many other factors. Magesh et al. [37] have proposed an AI-based algorithm for predicting COVID-19 cases using a hybrid Recurrent Neural Network (RNN) with a Long Short-Term Memory (LSTM) model. The authors have conducted their experiments while considering some demographic factors like sex, age, and temperature. Indeed, many other social factors were not considered in their model. Pinter et al. [20] have developed a hybrid machine learning approach to forecast COVID-19 cases in Hungary. The proposed hybrid approach encompasses the adaptive network-based fuzzy inference system and multi-layered perceptron-imperialist competitive algorithm. A machine learning-based approach for predicting COVID-19 new cases has been proposed in the study by Tuli et al. [38], who have used an iterative weighting for fitting Generalized Inverse Weibull distribution. For extensive study and more details about the forecasting approaches for COVID-19, the interested readers are referred to the work by Bragazzi et al. [21] who have reviewed the potentials of applying artificial intelligent and big data based approaches in predicting and managing the COVID-19 Pandemic outbreak. These previous studies show successful application of data analytics in multiple areas. Therefore, it is reasonable to use data analytics in this study.

From the above, it is clear that most of the data analytics studies have focused on historical data of confirmed COVID-19 cases, while some studies have considered some factors like temperature and patient sex. Indeed, many other important external factors that affect the spread of the disease have been completely ignored. These important factors include population, median age index, public and private healthcare expenditure, air quality as a CO_2_ trend, seasonality as month of data collection, number of arrivals in the country/territory, and education index. This results in a poor prediction of the number of COVID-19 cases.

## 3. Research Gaps and Contribution

A thorough examination of the literature reveals some observations, which can be outlined as follows. First, there is no previous study that simultaneously considers the historical data of the number of COVID-19 cases and most of the external factors that affect the spread of the virus. Secondly, there is no research work that provides future prediction of the number of COVID-19 cases using data analytics techniques. Therefore, efforts of the government to improve the healthcare system in the affected countries are greatly hampered. Consequently, in this research work, we have tried to fill this gap by proposing a data analytics algorithm, in which all the aforementioned features can be simultaneously considered.

This paper has the following contributions. Firstly, in contrast to the existing approach [17,18], which only focuses on the historical data of persons infected with COVID-19, we propose a more robust approach. Our approach simultaneously considers the historical data of COVID-19 cases alongside most of the external factors that affect the spread of the disease. These external factors include population, median age index, public and private healthcare expenditure, air quality as a CO_2_ trend, seasonality as month of data collection, number of arrivals in the country/territory, and education index. To consider all those massive number of factors, we develop a nonlinear autoregressive exogenous input (NARX) neural network-based algorithm. This algorithm is developed because it is the most appropriate one to handle time-based factors, like the number of COVID-19 cases. Moreover, NARX algorithms have been successfully applied in different research areas, as shown in Section 2.3.

Second, instead of predicting the number of COVID-19 cases in one or two countries [7,8,9], we use our algorithm to predict the number of COVID-19 in multiple countries, including top five affected countries in each continent. This is fruitful as it gives wide information about the spread of COVID-19 in different parts of the world.

Lastly, it has been observed in the literature that most research papers have not provided future prediction of the number of COVID-19 cases. As opposed to these previous research papers, we use the trained data produced from our algorithm to make future prediction of the number of COVID-19 cases. By using such predictions, both the government and people in the affected countries can take appropriate measures to resume pre-epidemic activities.

## 4. Data Analytics for Predicting New Daily Cases of COVID-19

In this section, we present how data analytics can be used in predicting the new daily cases of COVID-19. Instead of using the traditional approaches, which either focus on historical data or assume a normal distribution for the number of daily cases, we use a data analytics approach. In particular, this approach has the ability to consider a massive amount of data, including historical data of daily cases besides other external factors. The proposed methodology includes a nonlinear autoregressive exogenous input (NARX) neural network-based algorithm. The main steps of this algorithm are presented as follows:

Step 1: Collecting the data. The data have been collected from online websites, including “Worldometers” [39], “Our World in Data” [40], “World Bank Open Data” [41], and the official website of the World Health Organization (WHO). Besides, human development reports have been used to pick other kind of information, like median age and education index [42]. The scope of this study includes collecting data for about 189 countries/territories by focusing on two types of data: main data and other external factors. The main data include considering the number of confirmed coronavirus disease cases/day, the number of deaths due to coronavirus disease/day, and the total number of confirmed cases [39,40]. The external factors, on the other hand, include considering the factors that affect the spread of coronavirus disease. Note that the data have been collected for about 224 days, from 31 December 2019 until 10 August 2020. This leads naturally to set the size of data at 224.
Step 2: Preprocessing the data. While collecting the data, it was observed that data were not always available for the whole 189 countries/territories. To alleviate this situation, a refinement was performed by ruling out any countries/territories that suffer from data unavailability. This results in cancelling around 39 countries/territories, so that only 150 countries/territories have been considered. Our preliminary goal is to predict the new cases for all 150 countries/territories. However, this is not reasonable for two reasons. Firstly, it is not possible to present all the results in a single study due to page limitation. Secondly, it is computationally expensive to run this algorithm for 150 countries/territories. For the above reasons, we have limited our scope to considering the most affected countries in each continent. By doing so, the top five affected countries/territories have been considered from each continent. More details are presented in Section 5.
Step 3: Identifying the input sets. These sets contain historical data of some information alongside the external factors. These sets can be outlined as follows:
  - Main set, which includes two main information: the number of deaths due to coronavirus disease/day and the total number of confirmed cases;
  - External factor set that comprises the factors that affect the spread of coronavirus including population [42], median age index [41], public and private healthcare expenditure [41], air quality as a CO_2_ trend [42], number of arrivals in the countries/territories [41], and education index [42]. There is another factor that should be considered, called seasonality. Before incorporating this factor in the model, it should be clarified here that, in most countries, we can find cities with different seasons. For example, Iran has four seasons in its different cities [43]. Other examples include USA, China, Saudi Arabia, and Egypt. This observation indicates that, to consider the seasonality using seasons as a factor, the cities should be the scope of the study. Since the scope of this study is not cities but the countries, seasonality factor using seasons themselves cannot be considered in our algorithm. To find a compromise for this situation, the month of collecting data is selected to capture the seasonality in the proposed algorithm. It should be noted that the main data for the daily COVID-19 cases have been collected from the website “Our World in Data”, corrected through the website “Worldometers”. Next, the data have been doublechecked and refined by the data from the official website of WHO. In addition, because the considerable predictors are diverted, and they are not available on one database, their data have been collected from several websites. In further details, the data that have been collected from the website “World Bank Open Data” are the median age, number of arrivals, and health expenditure as a percentage of GDP [41], while the education index has been collected from United Nations Development Programme [42].
Step 4: Test of hypothesis using regression analysis. Since our study goal is to accurately predict the number of COVID-19 cases, we should focus on the most influential external factors. To do so, test of hypotheses using regression analysis should be conducted for each external factor. These hypotheses can be outlined as follows:

**Hypothesis** **#** **1.** $H_{0}:$ *The population has not a significant effect on the COVID-19 spread.* $H_{1}:$ *The population has a significant effect on the COVID-19 spread.*

**Hypothesis** **#** **2.** $H_{0}:$ *The median age index does not have a significant effect on the COVID-19 spread.* $H_{1}:$ *The median age index has a significant effect on the COVID-19 spread.*

**Hypothesis** **#** **3.** $H_{0}:$ *The public healthcare expenditure does not have a significant effect on the COVID-19 spread.* $H_{1}:$ *The public healthcare expenditure has a significant effect on the COVID-19 spread.*

**Hypothesis** **#** **4.** $H_{0}:$ *The private healthcare expenditure does not have a significant effect on the COVID-19 spread.* $H_{1}:$ *The private healthcare expenditure has a significant effect on the COVID-19 spread.*

**Hypothesis** **#** **5.** $H_{0}:$ *The air quality as a CO_2_ trend does not have a significant effect on the COVID-19 spread.* $H_{1}:$ *The air quality as a CO_2_ trend has a significant effect on the COVID-19 spread.*

**Hypothesis** **#** **6.** $H_{0}:$ *The number of arrivals in the countries/territories does not have a significant effect on the COVID-19 spread.* $H_{1}:$ *The number of arrivals in the countries/territories has a significant effect on the COVID-19 spread.*

**Hypothesis** **#** **7.** $H_{0}:$ *The education index does not have a significant effect on the COVID-19 spread.* $H_{1}:$ *The education index has a significant effect on the COVID-19 spread.*

**Hypothesis** **#** **8.** $H_{0}:$ *The seasonality as month of collecting data does not have a significant effect on the COVID-19 spread.* $H_{1}:$ *The seasonality as month of collecting data has a significant effect on the COVID-19 spread.*

After outlining the test of hypothesis, the regression analysis has been conducted, in which the *p*-value is calculated. If the *p*-value < 0.05, which is the significance level in this study, we reject the null hypothesis $H_{0}$ and go in favor of the alternative hypothesis $H_{1}.$ If p-value ≥ 0.05, we cannot reject the null hypothesis $H_{0}$. By doing so, the significant factors have been picked, including all the previous external factors except public health expenditure and air quality as a CO_2_ trend. More details about test of hypothesis using regression analysis are shown in Section 5.1.

Step 5: Designing the neural network structure. We have utilized the feedforward time-delay neural network as this structure has been commonly used in the literature due to its efficiency [44]. This network is composed of three main layers: input, hidden, and output. Regarding the activation function, the sigmoid function has been selected because it is efficient in reflecting the non-linear relationship among multiple factors.
Step 6: Training the neural network. To achieve this goal, the supervised learning method has been adopted. In this method, 70% of the data have been used for training purposes, whereas the rest have been reserved for validation and testing purposes.
Step 7: Predicting new cases of COVID-19. The trained data, known as the output of the network, have been used to predict the new cases of COVID-19 in the period from August 2020 until September 2020.

**Figure 3:**
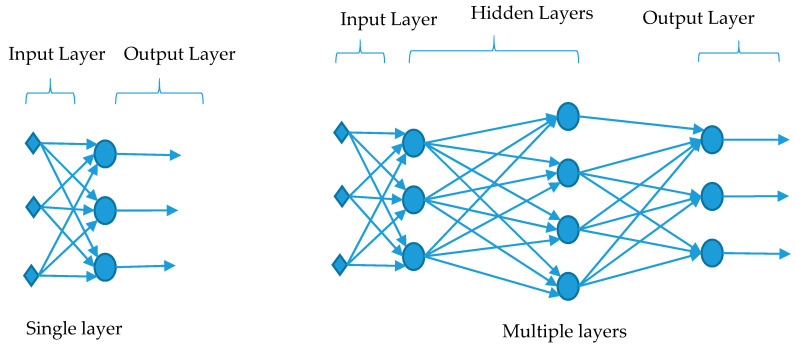
Structure of neural network.

Figure 3 represents diagrammatically the structure of the neural network. It should be noted that neural network is a common artificial intelligence technique. Indeed, this technique uses the idea of information flow between brain neurons, which is represented as a network via arrows and nodes. Arrows represent the input details and the output information, whereas nodes stand for the neurons. Usually, the nodes or neurons receive the input data, then analyze it to give suitable outputs. This straightforward movement of data from several input points is the simplest way to obtain an output. Such network structure is called feedforward neural network (FF), which has been used in our algorithm. Usually, feedforward neural network is either single layer feedforward neural network, as shown in the left-hand side of Figure 3, or multiple layers feedforward neural network, as shown in the right-hand side of Figure 3. In multiple layers feedforward neural network, the input layer is indirectly connected with the output layer by means of hidden layers (i.e., each layer in the network is in connection with the next layer). In particular, the input is connected to the first hidden layer, and this layer is connected to the next hidden layer. These connections move forward in this sequence until reaching to the output layer.

As mentioned earlier, the neural network structure adopted in this research is feedforward neural network with multiple layers. The type of the neural network is NARX neural network. The analysis of this network is based on the time-series modeling [45]. This means that it uses data obtained at successive times in the past in order to predict data in the future. Therefore, it is commonly used as a predicting tool in different fields, such as predicting the solar radiations per day [46], predicting electricity price of day-ahead [47], and the prediction of bearing life [48]. As any neural network, input data are processed in the NARX neural network through the nodes using the following function:

(1) $$a\left(t\right)=f\left(a\left(t-1\right),\;a\left(t-2\right),\;\ldots .,\;a\left(t-n_{a}\right),\;b\left(t-1\right).\;b\left(t-2\right),\;\ldots ,\;b\left(t-n_{b}\right)\right)$$

where a(t) is the output of the NARX neural network at time t. On the other hand, the values a(t−1),a(t−2), …., a(t−n) are the outputs of the NARX neural network in the past, whereas $n_{a}$ is the number of delays in the output. The values $b\left(t-1\right).\;b\left(t-2\right),\;\ldots ,\;b\left(t-n_{b}\right)$ are the inputs of NARX neural network, and $n_{b}$ is the delay in the inputs. From this equation, it is clear that, in order to get an output a(t) at time t, not only the input data is used but also the output data of the past should be used as well. For example, in order to predict the number of COVID-19 tomorrow a(t), the input data $b\left(t-1\right).\;b\left(t-2\right),\;\ldots ,\;b\left(t-n_{b}\right)$ are used. Besides, the predicted data of today and the past few days $a\left(t-1\right),\;a\left(t-2\right),\;\ldots .,\;a\left(t-n_{a}\right)$ will be used as well.

After presenting the algorithm, some questions might be asked. One of these questions is “is seasonality a factor that might have an impact on the results?”. Before answering this question, it should be clarified here that, in most countries, we can find cities with different seasons. For example, Iran has four seasons in its different cities [43]. Other examples include USA, China, Saudi Arabia, and Egypt. This observation indicates that, to consider the seasonality as a factor, the cities should be the scope of the study. Since the scope of this study is not cities but countries, we have considered the month of data collection as a measure of seasonality to overcome the above situation.

The proposed algorithm deals with variable population size, meaning that countries with higher population size impact more on the algorithm than lower population size countries, which can induce a high uncertainty in the predictions. The question here is “How this fluctuation was accounted for in the algorithm?” Indeed, to avoid the high fluctuation in the predictions, the best parameter setting for the algorithm should be used, while adopting the proposed algorithm in prediction [49]. For this purpose, the Taguchi method has been adopted, as shown in Section 5.2.

## 5. Experiments and Results

After presenting the NARX neural network-based algorithm that helps in predicting the new cases of COVID-19, it is necessary to present the effectiveness of the algorithm. For this purpose, some experiments are conducted while considering the top five affected countries from each continent, as shown in Table 1. Note that the experiments of this case study have been performed using an Intel i5 CPU and 2.52 GHz clock speed laptop. The memory is 8 GB RAM and runs the Windows 10 software. In addition, the algorithm is coded in MATLAB2019a. The results of experiments are presented in the following subsections.

### 5.1. Test of Hypothesis Using Regression Analysis

Before conducting the experiments of this study, we have collected the external factors that seems to affect the spread of coronavirus. These factors include population, median age index, public and private healthcare expenditure, air quality as a CO_2_ trend, seasonality as month of data collection, number of arrivals in the countries/territories, education index, and the month of collecting data. Since our study goal is to accurately predict the number of COVID-19 cases, only the most influential external factors should be considered. Towards this goal, the test of hypothesis using regression analysis has been adopted using Minitab software [32,50], in which the number of COVID-19 cases and their related external factors have been collected for about 160 countries/territories. Note that the regression analysis has been conducted with a significance level of 5% [32]. The results of hypothesis test are summarized in Table 2.

By looking at the results presented in Table 2, it is noticed null hypothesis $H_{0}$ related to Hypotheses # 1, 2, 4, 6, 7, and 8 is rejected, and the alternative hypothesis $H_{1}$ is picked. This means that the external factors like population, median age index, private healthcare expenditure, number of arrivals, education index, and month of collecting data have a significant effect on the number of COVID-19 cases. This is because p-values of these factors, which appear in boldface, are lower than the significance level, which is 5% in this study. In contrast, the null hypothesis $H_{0}$ related to Hypotheses # 3 and 5 cannot be rejected, meaning that alternative hypothesis $H_{1}$ is rejected. This indicates that external factors like public healthcare expenditure and CO_2_ trend do not have significant effect on the number of COVID-19 cases. Based on the above test of hypothesis, our experiments are further conducted while considering only the significant external factors, meaning considering all the factors except public healthcare expenditure and CO_2_ trend.

### 5.2. Parameter Settings of NARX Neural Network-Based Algorithm

After selecting the most influential factors, it is the time for conducting the prediction experiments. However, before doing so, the best parameter setting of NARX neural network-based algorithm should be determined. Towards this end, the most influential parameters are selected, and their corresponding levels are determined [33,51,52], as shown in Table 3. To select the best parameter settings, Taguchi method has been utilized as it is one of the effective tools in determining the best parameter settings by applying an orthogonal array and signal-to-noise (S/N) ratios [49,51,52,53]. The orthogonal array approach can be defined as an economic approach that is commonly adopted with an objective of minimizing the number of conducted experiments. The S/N ratio can be described as a performance indicator that indicates the quality of each conducted experiment. Since our Taguchi experiment includes four parameters with three levels, the orthogonal array $L_{9}$ should be selected in our experiments, which have been conducted using Minitab software.

**Figure 4:**
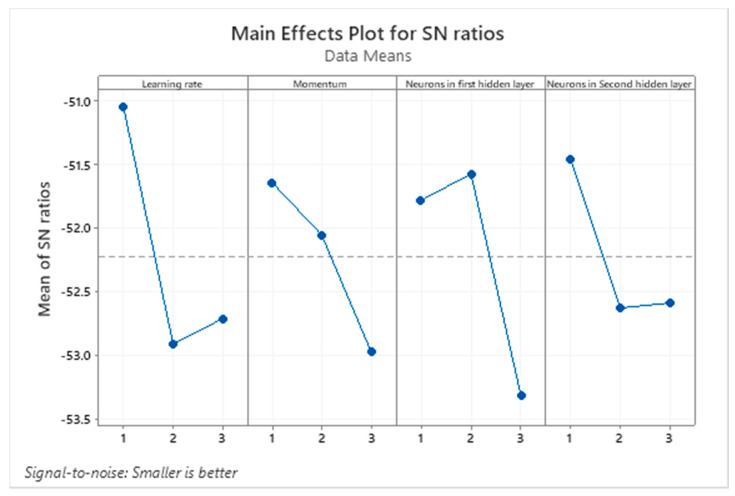
Main effect plot for S/N ratio while using the NARX neural network-based algorithm.

Figure 4 illustrates the average S/N ratio of the selected parameter at each level, while using our proposed algorithm. Since our algorithm aims at predicting the COVID-19 cases, the objective in this study is to minimize the error between the predicted and real values. Based on this observation, the parameter level should be selected based on the smaller is better criterion. This means that the level with small average S/N ratio is better than the level with higher average S/N ratio. By applying this criterion in Figure 4, the best level for the parameters 1, 2, 3, and 4 should be set at are levels 2, 3, 3, 2, respectively. These levels appear in a boldface in Table 3.

### 5.3. Performance of the NARX Neural Network-Based Algorithm

In this section, we report the performance of the NARX neural network-based algorithm. It should be noted that the performance of the algorithm has been evaluated using a commonly used performance indicator, called root mean square error (RMSE) [44]. The RMSE is used to reflect the error between real and predicted values of COVID-19 cases. Besides, the correlation has been calculated to indicate the closeness of the predicted data to observed data. To select the suitable correlation test, the normality of the observed and predicted COVID-19 cases should be checked. By doing so, it has been noticed that both the observed and predicted COVID-19 cases are not normally distributed. This observation naturally leads to using Spearman correlation test [54,55,56]. To measure the model uncertainty, the error standard deviation has been calculated [57]. Details of the results are presented in Table 4.

By looking at Table 4, it is noticed that the value of the RMSE is low in most African and Asian countries. This is because the values of predicted and real cases are a bit low if compared with other countries. It is also observed that that the value of the RMSE is a bit large in some countries like USA, Spain, and China. For instance, RMSE = 420 while considering USA. At a first glance, an RMSE value of 420 can give the impression of a large difference between predicted and real values, implying a poor performance of the proposed algorithm. Indeed, 420 is not that big at all, because predicted or real values reach up to 48,529. Thus, 420 is not a big figure if compared with 48,529, meaning that the performance of the algorithm is still reasonable, while handling a large number of COVID-19 cases. To summarize, we can say that the proposed algorithm produces large RMSE when the real and predicted values are large, and vice versa. This indicates the consistency and robustness of the proposed algorithm.

Regarding the correlation, it is observed that the correlation factor is larger than 0.9 in all countries with *p*-value of zero. This means a strong positive significant correlation between the observed and predicted data, which indicates a closeness of the predicted data to the observed data. This reflects the high accuracy of the proposed algorithm. By looking at the error standard deviation, it indicates low error variability in countries characterized with low number of COVID-19 cases and vice versa. This confirms the stability and reliability of the proposed algorithm.

### 5.4. Performance Analysis

After presenting the performance of the proposed algorithm, there is a question that might be asked here, “what is the advantage of the proposed algorithm over the existing traditional method in the literature?”. To answer this question, our experiments have been further extended to make a comparison between our proposed algorithm and the traditional method that can be represented in the study by Chen et al. [17]. Note, both studies have the same objective, which is predicting the number of COVID-19 cases. However, both studies are different in their considered factors. The study by Chen et al. [17] has only focused on historical data of the number of COVID-19 cases while considering a limited number of factors, like travel and occupation. In contrast, our study has the same focus as the study Chen et al. [17], besides, it has considered many external factors that overlooked in their study. These factors include population, median age index, public healthcare expenditure, private healthcare expenditure, air quality as a CO_2_ trend, education index, and seasonality as month of collecting data. The experiment results obtained from both approaches are summarized in Table 5.

By looking at Table 5, the results show that the NARX neural network-based algorithm is more accurate than the traditional method. This outperformance is due to considering more factors that affect the spread of COVID-19, such as the external factors like the population, the health expenditures, and others. This results in an accurate prediction for the proposed algorithm. In contrast to the proposed algorithm, the traditional method only focusses on the historical data and neglect many external factors. Hence, some important factors that affect the spread of the virus are neglected, leading finally to a poor prediction of the number of COVID-19 cases.

This section establishes that the proposed algorithm gives improved results when compared with the traditional method. Thus, the significance of utilizing this algorithm in real practice is further affirmed.

### 5.5. What Next in the Future?

**Figure 5:**
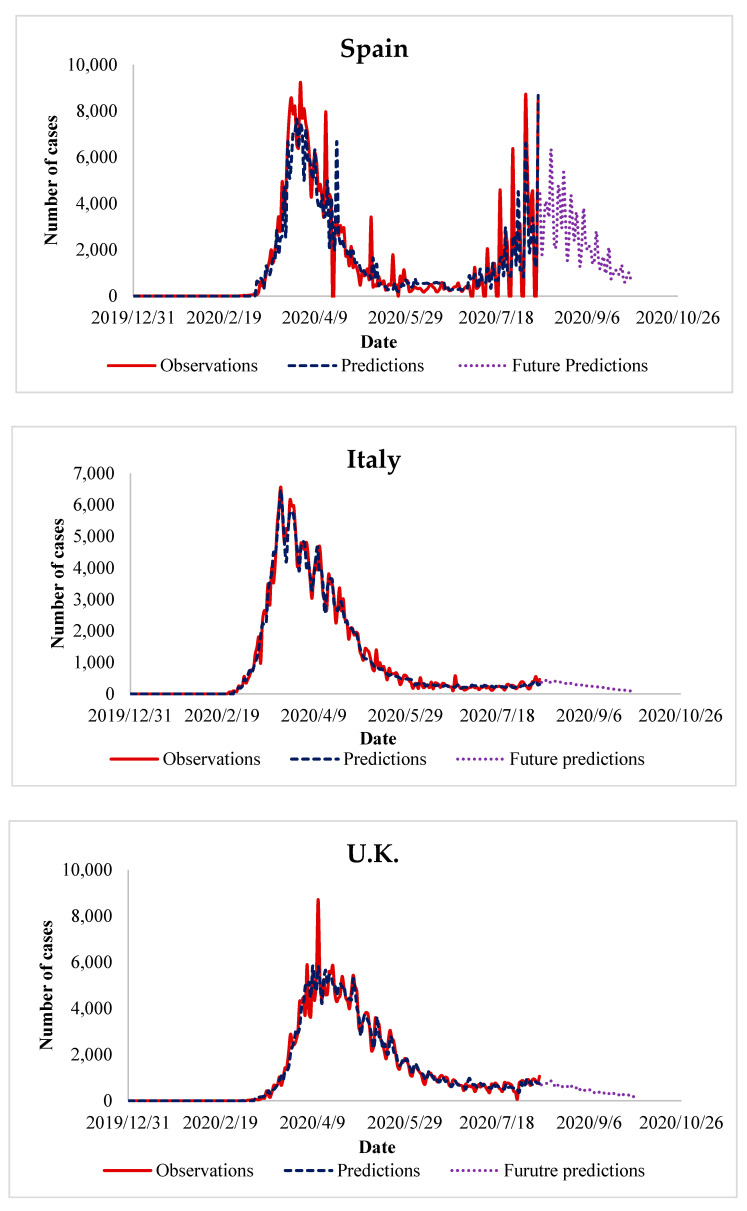

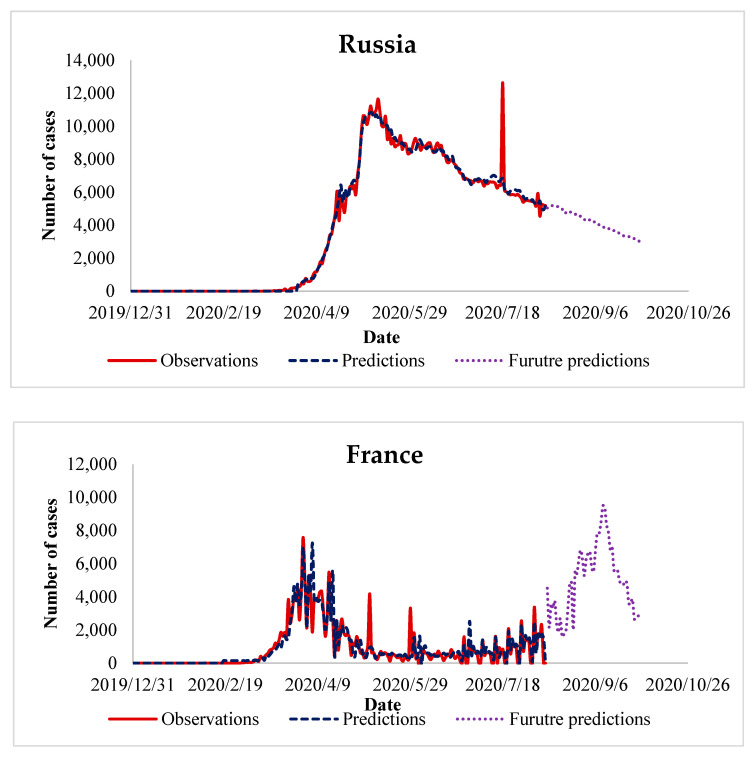
Future prediction of COVID-19 cases in European countries.

**Figure 6:**
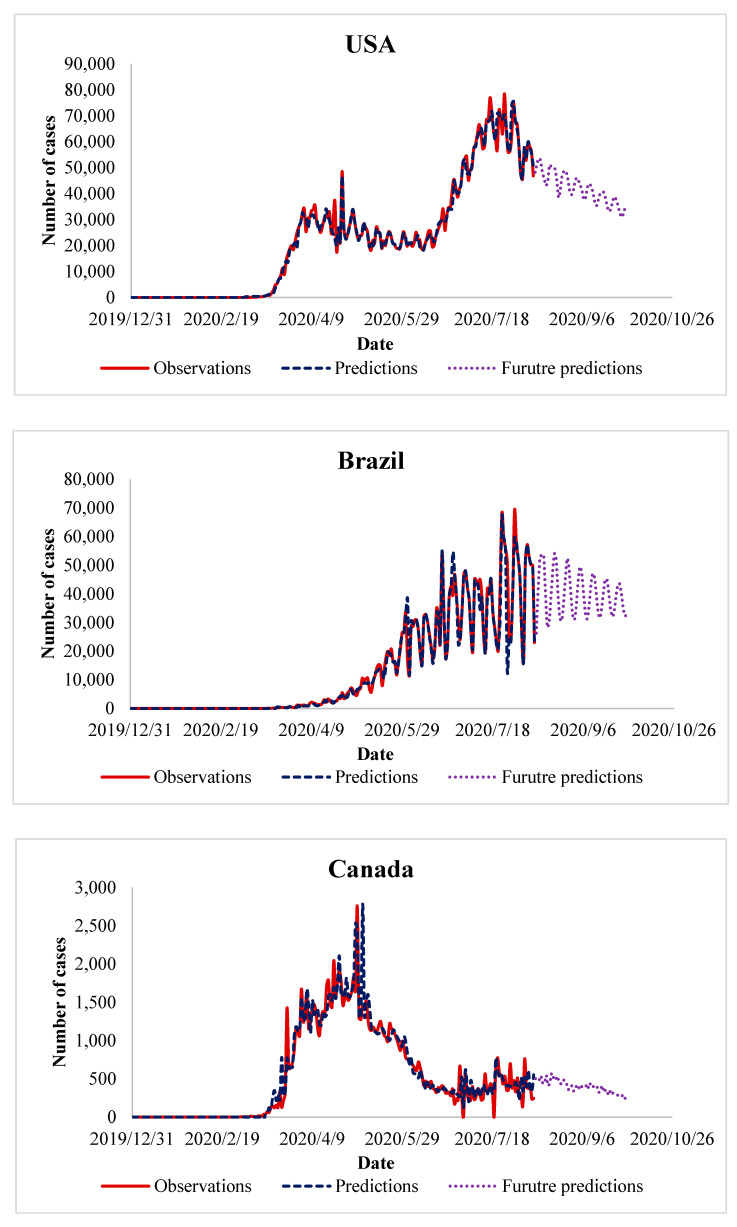

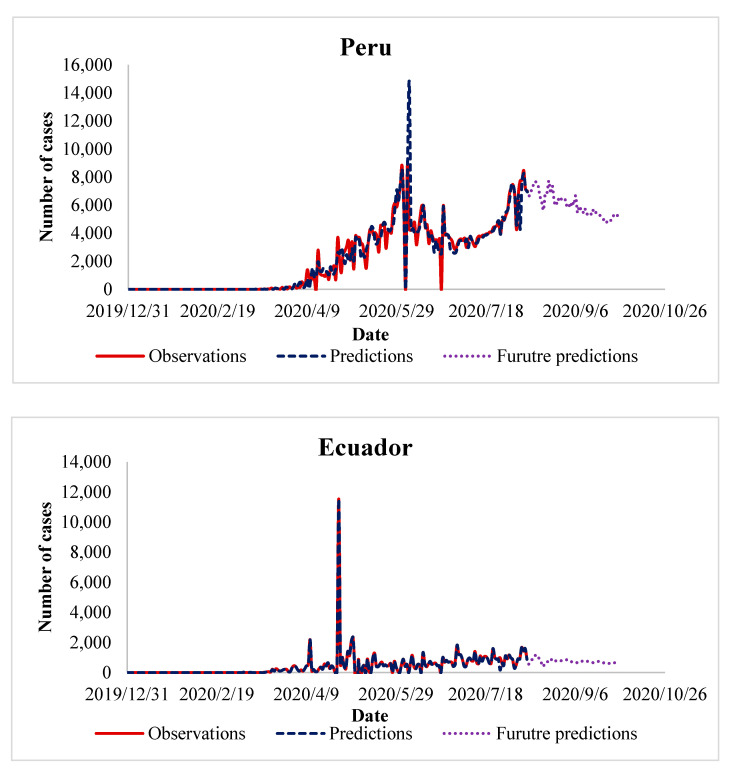
Future prediction of COVID-19 cases in North and South American countries.

**Figure 7:**
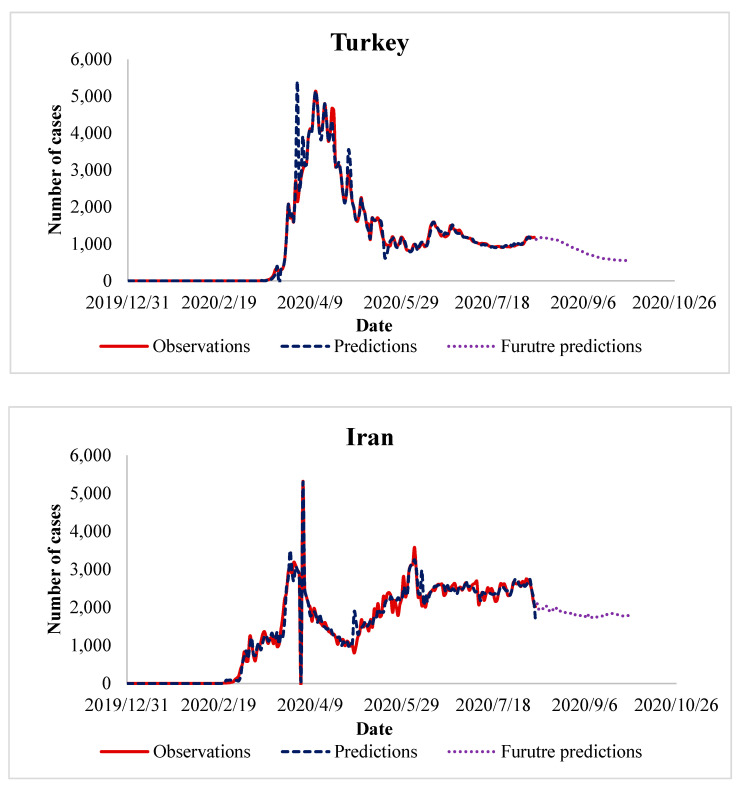

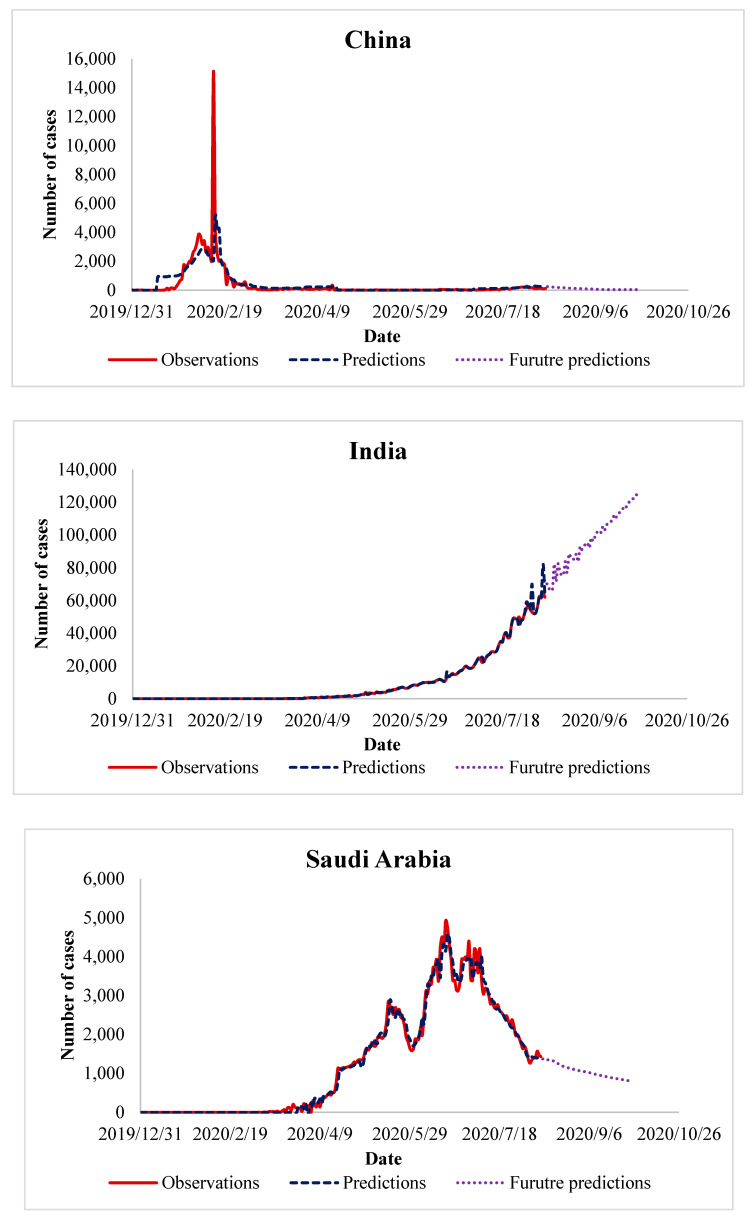
Future prediction of COVID-19 cases in Asian countries.

**Figure 8:**
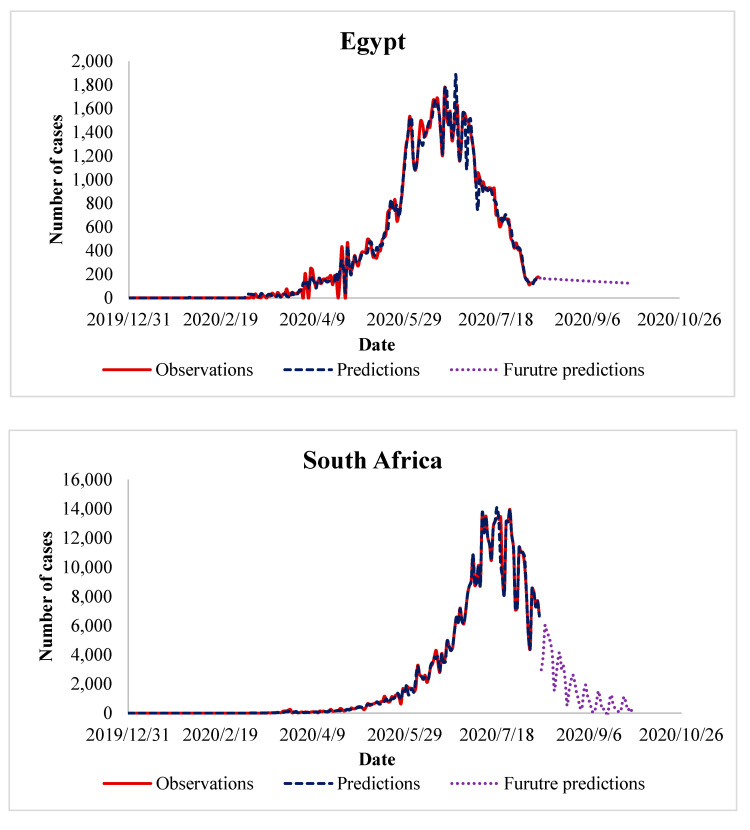

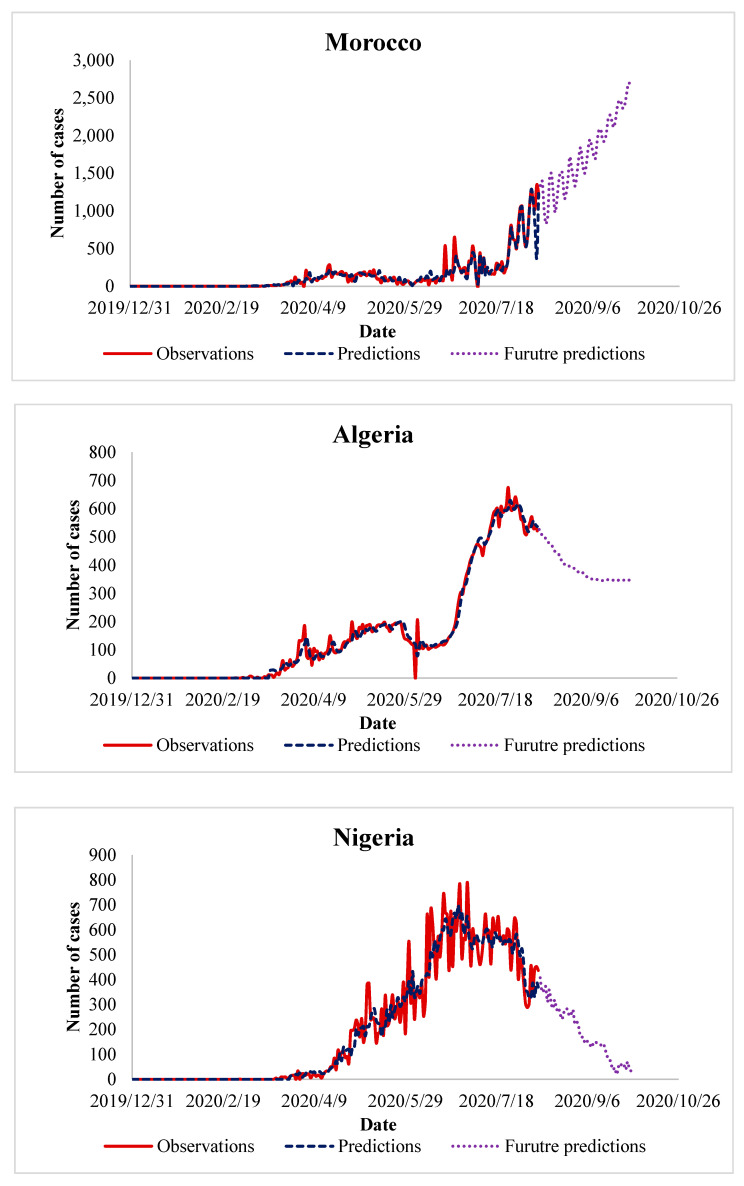
Future prediction of COVID-19 cases in African countries.

So far, we have presented the performance of the proposed algorithm and its advantage over the existing methods. It is fine, but still, there are some questions that have not been answered like “what next in the future?”, “how can we benefit from the algorithm in predicting future cases of COVID-19?”, “when will COIVD-19 end?”. Answering these questions necessitates extending our experiments, in which we use the trained data to predict the number of future cases of COVID-19. In the experiments of the previous sections, we observe that in the countries that control the spread of COVID-19, the peak in the number of daily cases appeared after 3–4 months. This observation has been taken as a reference to predict future COVID-19 cases in the countries where the disease is yet to peak. It is interesting to recall that the future prediction has been done for about two months, in the period from August 2020 until September 2020. The results of these experiments are presented in Figure 5, Figure 6, Figure 7 and Figure 8, which represent the future predictions in Europe, North and South America, Asia, and Africa, respectively.

After presenting the future prediction of COVID-19 cases in European countries, we have some observations, which are outlined as follows:

- In most European countries, like Italy, UK, and Russia, the number of cases has already reached its peak before our future prediction. Based on this observation, we predict that the number of future COIVD-19 cases will decrease gradually during the period from August 2020 until September 2020. It is worth to mention that our predicted reduction in the number of COVID-19 cases appear during August 2020. The abovementioned reduction is because of strict compliance with the precaution guidelines established by WHO.
- In contrast to most of European countries, the situation in Spain and France is quite similar, as the number of cases has raised recently and formed another peak. In Spain, the second peak has been already formed, therefore, our algorithm predicts a gradual decrease during the period from August 2020 until September 2020. In France, the algorithm predicts a slight increase followed by a gradual decrease in the number of cases during the same period.

By looking at Figure 6, some observations can be summarized as follows:

- In the case of USA, the number of COVID-19 cases has already formed its second peak by mid of July 2020. Therefore, we have predicted a slow reduction in the number of future COVID-19 cases. This prediction, in terms of reduction, appears in the USA during the first half of August 2020. It should be noted that this slow reduction is because of the dysfunction experienced in the healthcare system of the USA [59].
- In the case of Brazil, we observe that the peak has been reached. Then, we predict that the number of future COVID-19 cases will experience a wavy reduction during the period from August 2020 until September 2020. It is important to mention that the predicted slow reduction in future COVID-19 cases agrees with the actual reduction realized during August 2020. This wavy reduction is due to overlooking the social distancing instructions by most of Brazilian residents [60].
- In case of Canada, the number of COVID-19 cases has reached its peak since beginning of May 2020. Therefore, it is reasonable to predict a gradual decrease in the number of cases during the period from August 2020 until September 2020.
- In the case of Peru, we observe an increase in the number of COVID-19 cases by July 2020. Then, we predict that, during the period from August 2020 until September 2020, this increase will continue a bit before a decline in the number of future COVID-19 cases. This increase is due to the bad behavior of the people, so that the situation becomes even worse during those days [61].
- In the case of Ecuador, we predict the number of future COVID-19 cases will keep its wavy motion, meaning that the number will tend to zero and increase again. This wave appears because there is no transparency in the reported number of COVID-19 cases, meaning that the government has not disclosed the real number of COVID-19 cases [62].

After presenting the results of Asian countries, we have the following observations:

- China is one of the few cases that has fully controlled the situation. This is apparent as the number of future COVID-19 cases is almost zero. Thanks to the Chinese government and medical system, strict typical quarantining measures have been implemented, thus, leading finally to overcoming this hard time.
- The situation in Turkey, Iran, and Saudi Arabia is like other European countries that have reached the peak. Our algorithm predicts a gradual reduction in the number of future COVID-19 cases, which has been realized during August 2020.
- The situation in India is completely different compared to the rest of Asian countries. This is because India is yet to reach its peak. Based on this observation, we predict that the increase in the number of COVID-19 cases will continue during the period from August 2020 until September 2020.

By looking at Figure 8, we can draw the following observations:

- Most of African countries are quite similar, except Morocco, as the peak of COVID-19 cases has already appeared. It is predicted that the future number of COVID-19 cases will decrease gradually, during August and September 2020. So far, the trend of our predicted graph has been realized in the aforementioned African countries.
- In contrast to the above-mentioned African counties, in Morocco, the peak of COVID-19 cases has not yet appeared. Therefore, the future number of COVID-19 cases will continue its increase during August and September 2020. It is important to mention that this increase has been realized during the first half of August 2020.

Based on the above observations, we outline some recommendations, which are as follows:

- It is recommended for the people living in the USA, Brazil, Ecuador, Peru, and India to strictly follow the precautions instruction recommended by WHO. This includes quarantining infected people, whereas healthy people should stay home to avoid COVID-19 infection, and when they go out, they should follow the rules of social distancing.
- It is recommended for the government and healthcare system of countries like the USA and Brazil to raise their private and public health expenditures to control the number of future COIVD-19 cases. In addition, penalties may be applied to the people who violate the instructions recommended by WHO.
- It is recommended for the government of Ecuador to release the correct number of COVID-19 cases so that the people can understand the severity of the situation and obey the health guidelines released by WHO.
- In the countries that fully or partially control the COVID-19 like China, it is recommended for the people to keep following the medical instructions. Otherwise, COVID-19 may come back in a mutated form causing another global pandemic.

## 6. Conclusions

This study investigates how new COVID-19 cases can be predicted while considering the historical data of COVID-19 cases alongside the external factors that affect the spread of the virus. To do so, data analytics was adopted by developing a nonlinear autoregressive exogenous input (NARX) neural network-based algorithm. The effectiveness and superiority of the developed algorithm are demonstrated by conducting experiments using data collected for top five affected countries in each continent. The results show an improved accuracy if compared with the existing methods. Moreover, the experiments are extended to make future prediction of the affected COVID-19 cases during the period from August 2020 until September 2020. The predicted COVID-19 cases help in providing some recommendations for both the government and people of the affected countries.

This study provides a novel way for predicting the number of COVID-19 cases. However, there are some venues that might be suitable for future directions. For example, predicting the number of deaths could be one direction. Another direction might be predicting the number of recovered people. One of the fruitful ideas is predicting the number of COVID-19 cases in the top affected cities, while considering the seasonality factor.

## Figures and Tables

**Table 1:** Top five affected countries in each continent.

Continent	Country
Europe	Spain, Italy, UK, Russia, and France
North and South America	USA, Brazil, Canada, Peru, and Ecuador
Asia	Turkey, Iran, China, India, and Saudi Arabia
Africa	Egypt, South Africa, Morocco, Algeria, and Nigeria

**Table 2:** Results of regression analysis.

Hypothesis	*p* Value	Decision	Interpretation
Hypothesis # 1: population	**0.000**	Reject $H_{0}$ and pick $H_{1}$	Population is significant
Hypothesis # 2: median age index	**0.041**	Reject $H_{0}$ and pick $H_{1}$	Median age index is significant
Hypothesis # 3: public healthcare expenditure	0.523	Cannot Reject $H_{0}$ and reject $H_{1}$	Public healthcare expenditure is not significant
Hypothesis # 4: private healthcare expenditure	**0.000**	Reject $H_{0}$ and pick $H_{1}$	Private healthcare expenditure is significant
Hypothesis # 5: air quality as a CO_2_ trend	0.476	Cannot Reject $H_{0}$ and reject $H_{1}$	Air quality as a CO_2_ trend is not significant
Hypothesis # 6: number of arrivals in the countries/territories	**0.000**	Reject $H_{0}$ and pick $H_{1}$	Number of arrivals in the countries/territories is significant
Hypothesis # 7: education index	**0.047**	Reject $H_{0}$ and pick $H_{1}$	Education index is significant
Hypothesis # 8: seasonality as month of collecting data	**0.000**	Reject $H_{0}$ and pick $H_{1}$	Seasonality as month of collecting data is significant

**Table 3:** Levels for the NARX neural network- based algorithm.

Parameter	Level 1	Level 2	Level 3
Learning rate	0.01	**0.1**	0.3
Momentum	0.1	0.3	**0.5**
Number of neurons in the first hidden layer	N/2=112	N=224	**1.5 × *N* = 336**
Number of neurons in the second hidden layer	0	***N*/2 = 112**	N=224

N: it equals the number of input data, which is 224 in this study.

**Table 4:** Results of the NARX neural network-based algorithm.

Continent	Country	NARX Neural Network-Based Algorithm			
		Root Mean Square Error (RMSE)	Spearman Correlation		Error Standard Deviation
			Correlation Factor	*p*-Value	
Europe	Spain	300	0.9687	0.000	902.74
	Italy	71	0.9725	0.000	215.38
	UK	113	0.9770	0.000	343.13
	Russia	150	0.9748	0.000	420.57
	France	189	0.9753	0.000	587.11
North and South America	USA	786	0.9882	0.000	23,792.25
	Brazil	1146	0.9828	0.000	3463.52
	Canada	54	0. 9566	0.000	163.93
	Peru	148	0.9716	0.000	423.08
	Ecuador	18	0.9712	0.000	57.03
Asia	Turkey	78	0.9753	0.000	251.77
	Iran	56	0.9784	0.000	170.81
	China	14	0.9319	0.000	34.36
	India	180	0.9946	0.000	550.63
	Saudi Arabia	56	0.9720	0.000	171.79
Africa	Egypt	20	0.9696	0.000	61.55
	South Africa	79	0.9847	0.000	241.57
	Morocco	28	0.9461	0.000	85.74
	Algeria	7	0.9740	0.000	22.87
	Nigeria	19	0.9695	0.000	57.61

**Table 5:** Comparison of the results obtained from the NARX neural network-based algorithm and traditional methods.

Continent	Country	RMSE of NARX Neural Network-Based Algorithm	RMSE of Traditional Methods	Improvement of NARX over Traditional Method (%)
Europe	Spain	300	49,492	99.39
	Italy	71	257	72.38
	UK	113	988	88.56
	Russia	150	371	59.59
	France	189	1973	90.42
North and South America	USA	786	15,840	95.04
	Brazil	1146	3891	70.55
	Canada	54	162	66.64
	Peru	148	2229	93.36
	Ecuador	18	777	97.68
Asia	Turkey	78	336	76.79
	Iran	56	152	63.10
	China	14	334	95.81
	India	180	615	70.73
	Saudi Arabia	56	140	60.07
Africa	Egypt	20	32	37.07
	South Africa	79	149	46.98
	Morocco	28	45	38.10
	Algeria	7	12	39.78
	Nigeria	19	27	28.76

Improvement (%) = $(RMSE_{Traditional}-RMSE_{NARX})\times 100/RMSE_{Traditional}$ [58].
